# Treatment with Magnesium in Pregnancy

**DOI:** 10.3934/publichealth.2015.4.804

**Published:** 2015-12-10

**Authors:** Ragnar Rylander

**Affiliations:** BioFact Environmental Health Research Centre

## 1. Introduction

With the growing understanding of the importance of a proper magnesium homeostasis during pregnancy, an increasing number of studies have used magnesium for treatment or prevention of different pathological conditions in pregnancy. The aims of the review is to describe the major studies performed on treatment with magnesium in pregnancy related complications. The studies reviewed were collected from a previous Cochrane review with differences in the criteria for the evaluation of the relevance of the studies [1].

## 2. Treatment

Based in the knowledge regarding the pivotal role of magnesium in enzyme systems, muscular function and bone calcium homeostasis [2], a large number of studies has been reported on the treatment of different pathological conditions with magnesium. Regarding pregnancy, the treatment of pre-eclampsia and eclampsia with MgSO_4_ is an established procedure. The treatment is initiated on suspicion of eclampsia or when the symptoms are fully established. It comprises an intravenous injection (usually 4–6 g), followed by sometimes followed by a maintenance dose given intravenously or intramuscularly. Side effects are nausea, flushing, and muscle weakness. A Cochrane review evaluated 13 studies, one of which comprised 10^ˆ^141 women [3]. In general the treatment was found to be efficient in about half the cases. Similar findings were reported in a review comprising studies from Bangladesh, India, Pakistan, Nigeria, and the UK [4].

The initial concept for introducing MgSO_4_ in the treatment of eclampsia and pre-eclampsia was as an anticonvulsive agent, as the anti-cramp properties of this agent (Epsom salt) have been known since the 1600^th^ century. Recent data suggest that the effect may be mediated through prevention of oxidative damage through interactions with alkyl radicals [5]. A study on pregnant women from Sub-Saharan Africa evaluated the role of MgSO_4_ treatment and increased use of transfers to hospitals [6]. About 17000 maternal deaths were associated with PE/E. A more extensive use of MgSO_4_ would have prevented 610 deaths.

In addition to treatment of pre-eclampsia and eclampsia, MgSO_4_ has also been used in attempts to protect the children from neurological disorders as reviewed in two meta-analysis [7],[8]. One study found a reduction of hypotension treatment and invasive mechanical ventilation in pre-term infants treated with MgSO_4_ [9].

## 3. Prevention

In addition to treating an established or almost established disease, several studies have attempted to avoid the development of disease by giving magnesium as a supplement during the whole pregnancy. The rationale for this is the many reports that describe an insufficient supply of magnesium in the food intake among pregnant women [10]–[12].

The conductance and interpretation of results from such magnesium supplementation studies in populations may be influenced by a number of methodological problems [13]. An effect of the supplementation can only be expected in a risk group i.e. persons with a magnesium deficiency. The proportion of such risk persons in the population studied or differences in that proportion between different population groups would thus influence the results. Evaluating the effect in the whole population studied without identifying the risk group might thus lead to a false conclusion concerning the absence of an effect.

The therapeutic range is of importance. Some earlier studies used low doses of magnesium in the supplementation—128 mg [14]. Another study administered magnesium (100 mg) also to the control group, yielding a therapeutic range of 270 mg [15]. This is relatively low in comparison to the 300–400 mg doses usually used. The lower dose administered to the control group—100 mg— might also have been sufficient to reduce a negative effect induced by magnesium deficiency.

### 3.1. Prevention—blood pressure

Several studies have investigated the possibility to prevent the pregnancy induced high increase in blood pressure, including pre-eclampsia and eclampsia, during pregnancy by giving a supplementation of magnesium throughout pregnancy. In a double blind, randomised controlled (DBRC) study in Denmark, 50 women with a pregnancy induced hypertension were given intravenous magnesium chloride for two days and then an oral supply of magnesium hydroxide till the end of the pregnancy [16]. In the supplemented group, mean atrial blood pressure was significantly lower from day three of supplementation till delivery.

In a DBRC study from China, 51 pregnant women were given 175 mg magnesium gluconate daily [17]. In the supplemented group 4% of the women developed hypertension as compared to 16% in the placebo group. In a partial DBRC study from Angola 150 pregnant women were given primrose and fish oil, magnesium oxide or placebo [18]. In the magnesium group there were two women with high blood pressure (systolic >30 and/or diastolic > 15 mm Hg) as compared to 13 in the untreated group.

In a DBRC study from Sweden, prima-para subjects were given 300 mg magnesium as a citrate salt or placebo from around pregnancy week 25 throughout the pregnancy [19]. The changes in diastolic blood pressure at different times during the pregnancy in relation to the value at 12 weeks were evaluated and magnesium sensitive transporter gene (MgTG) expressions were measured [20]. The percentage of women with an increase of ≥15 mm Hg in diastolic blood pressure was significantly lower in the magnesium supplemented group. Among those with an increase in urinary magnesium excretion during pregnancy, reflecting the increased intake, there was a decrease in the diastolic blood pressure (corr coeff -0.438, *p* = 0.005). Systolic and diastolic blood pressures at week 37 (excluding the magnesium treated group) were significantly related to the expression of the MgTGs *SLC41A1* and *SLC41A3*. In week 12 as well as in week 37, the expression of *TRPM6* was significantly higher as compared to a group of non-pregnant subjects (*p* = 0.007). In the placebo group the expression of gene *TRPM6* was significantly higher among those with an increase in diastolic blood pressure ≥15 mm Hg (0.84/0.07 vs. 0.64/0.04, *p* = 0.013). There was also an inverse relationship between the expression of *TRPM6* and the urinary excretion of magnesium (corr coeff -0.596, *p* = 0.015).

One study could not demonstrate an effect of magnesium- aspartate hydrochloride on high blood pressure [15]. This was not, however, a double blind study but administered two dose levels of magnesium—100 mg and 365 mg. It is likely that the narrow therapeutic range (265 mg) or the absence of a non-intervention group, reflecting basic conditions, decreased the sensitivity of the study. Another study could also not demonstrate an effect after administration of 360 mg magnesium hydrochloride throughout pregnancy [21]. Blood pressure data were, however, only presented for the whole group. The statistically significant differences in birth weight etc (see below) were only present when patients not taking their magnesium tablets properly were excluded. Blood pressure data from this group were not reported.

In summary, four studies from four different countries demonstrate that the supplementation with magnesium during pregnancy reduces the risk of pregnancy induced high blood pressure

### 3.2. Prevention – other effects

There are several studies where the effect of magnesium supplementation on other pregnancy complications than high blood pressure have been studied.

In one DBRC study with 985 pregnant women, the supply of 360 mg magnesium aspartate significantly reduced the frequency of premature deliveries (< 258 days), and the number of children with a birth weight < 500 g [22]. The incidence of pre-eclampsia was significantly lower in the magnesium treated group. Another DBRC supplementation study comprised 568 pregnant women, allocated magnesium hydrochloride (365 mg) or placebo according to even/odd numbers of birth date [23]. The number of days spent in hospital due to pregnancy complications was 533 days in the magnesium group and 887 days in the placebo group. The number of women with pre-term labour, pre-term deliveries, and incomplete cervix closure was significantly lower in the magnesium group. The effect on pre-eclampsia could not be evaluated due to the small number of subjects with this complication.

Contradictory findings were reported in a DBRC study on 374 pregnant women [15]. They received 375 mg magnesium/day in terms of magnesium-aspartate hydrochloride. No significant differences were found between the magnesium and placebo groups regarding blood pressure, foetal growth retardation, pre-eclampsia, preterm labour, birth weight, gestational age at delivery, or number of infants admitted to a special care hospital unit. The absence of any of these effects, quite in contrast to the previously cited study, could be due to an erroneous study design. The placebo group received a vitamin supplement containing 100 mg magnesium. Even this small dose might have influenced the investigated parameters, decreasing the difference between the magnesium high dose and the magnesium low dose groups to non-significant levels.

The same endpoints as in the above two studies were investigated in an investigation in Italy [23]. 100 pregnant women participated in this DBRC study where 360 mg magnesium was administered daily. There were fewer hospitalisation days for the mother in the supplemented group as well as fewer premature births, and less underweight (< 2500 g) children.

Similar findings were reported in a DBRC study on 240 magnesium citrate supplemented women and 250 controls [24]. The supplemented group had a significantly lower number of hospitalisation days for mothers due to threats of preterm labour, and higher birth weight for the babies. There was a tendency to lower rates of preterm labour and low birth weight (< 2500 g) in the supplemented group.

In a small study on pregnant women, selected for risk of pre-mature labour, 22 women received 216 mg magnesium gluconate [25]. There were no differences in terms of preterm labour, gestational age at delivery or mean birth weights. There was no magnesium serum level increase in the supplemented group among the five women with preterm labour.

Hypoxic, ischaemic encephalopathy (HIE) was studied in a sample of 4494 women with a low magnesium intake in food [26]. The supplementation was 64 mg magnesium/day, leading to a significantly higher level of red blood cell content of magnesium at delivery (1.75 vs 1.68 mmol/L). There was a significantly lower number of stillbirths in the magnesium supplemented group. There were no differences in gestational hypertension, premature onset of labour, or duration of pregnancy. There was a non-significant trend to a lower incidence of HIE in the magnesium treated group (15 vs 22 babies). If the supplemented and control groups were amalgamated, women with a red blood cell magnesium level below the median, had a sevenfold risk of having a baby with HIE. The authors concluded that due to the low incidence of HIE the study was statistically underpowered. The dose of magnesium in this study was quite low.

Leg cramps is a common symptom in magnesium deficiency and they become more prevalent during pregnancy. One study comprised 74 pregnant women with leg cramps [27]. The supplementation in this DBPC study was 360 mg magnesium as citrate or lactate. There was a significantly larger reduction of subjectively experienced stress due to leg cramps in the supplemented group. Persisting symptoms the days after night cramps were also lower.

## 4. Comments

It is beyond doubt that magnesium plays an important role for the functioning of many different organs in the body, particularly muscles [2]. Regarding supplementation with magnesium during pregnancy to decrease the incidence of pregnancy complications, the picture is less clear. For all effects studied, there are positive as well as negative outcomes. The Cochrane report examined the different studies and classified some of those showing a beneficial effect as “low quality studies” [1]. Applying the latest scientific paradigms concerning study designs as in that review, will obviously discard older studies, performed according to principles used at that time although the data may be valid. On the other hand it is important to perform a quality assurance, both regarding conclusions how to deal with the supplementation problem today and as a background for future studies, There are, however, some basic errors in the Cochrane evaluation. One study was discarded as low quality as the assignment of supplementation/placebo was made according to the mothers' birthday (odd-even) [21]. Even if this criterion does not fulfil the definition of DBRC as used today, the judgement is erroneous from a biological point of view. Birth dates, related to days, could not induce an error in relation to a biological effect. Furthermore, a study classified as good quality [15] administered magnesium also to the control group which is not a correct DBRC design and might have introduced dosage errors as discussed above.

Apart from the strict scientific judgement of the studies as in Cochrane [1], there must also be a personalised interpretation of available data and the risk related to supplementation, weighing information regarding possible benefits. In contrast to medication e.g. with aspirin which has been tried in pre-eclampsia [28], magnesium is a natural substance and completely harmless except for occasional cases of diarrhoea. This implies that a decision to prescribe supplementation with magnesium should also take into account data from studies which may not be perfect according to modern standards. One should also weigh the tremendous savings that would result in terms of suffering of children and mothers as well as the costs for caretaking and treatment of PE and E. This being said, there is obviously a need for further studies, particularly in risk groups with high incidences of PE and E. With the data available and the lack of a risk supplementing with magnesium, one could, however, question whether a double-blind design in such studies is ethically correct.

From a practical point of view, the absence of a consensus regarding magnesium treatment should not hinder the physician or maternal health care responsible to suggest a supplementation with magnesium, particularly to women with early symptoms such as leg cramps, oedema, and blurred vision.
